# Exploring the Dilemma of AI Use in Medical Research and Knowledge Synthesis: A Perspective on Deep Research Tools

**DOI:** 10.2196/75666

**Published:** 2025-07-15

**Authors:** Ariel Yuhan Ong, David A Merle, Siegfried K Wagner, Pearse A Keane

**Affiliations:** 1 UCL Institute of Ophthalmology, Institute of Ophthalmology, University College London, 11-43 Bath St, London, EC1V 9EL, United Kingdom, +44 7482213973; 2 Moorfields Eye Hospital, Moorfields Eye Hospital NHS Foundation Trust, London, United Kingdom; 3 NIHR Moorfields Biomedical Research Centre, London, United Kingdom

**Keywords:** artificial intelligence, AI, medical research, scientific writing, large language model, LLMs, hallucination

## Abstract

Advances in artificial intelligence (AI) promise to reshape the landscape of scientific inquiry. Amidst all these, OpenAI’s latest tool, Deep Research, stands out for its potential to revolutionize how researchers engage with the literature. However, this leap forward presents a paradox; while AI-generated reviews offer speed and accessibility with minimal effort, they raise fundamental concerns about citation integrity, critical appraisal, and the erosion of deep scientific thinking. These concerns are particularly problematic in the context of biomedical research, where evidence quality may influence clinical practice and decision-making. In this piece, we present an empirical evaluation of Deep Research and explore both its remarkable capabilities and inherent limitations. Through structured experimentation, we assess its effectiveness in synthesizing literature, highlight key shortcomings, and reflect on the broader implications of these tools for research training, and the integrity of evidence-based practice. With AI tools increasingly blurring the lines between knowledge generation and critical inquiry, we argue that while AI democratizes access to knowledge, wisdom remains distinctly human.

## Introduction

Advances in artificial intelligence (AI) are rapidly transforming the world across diverse fields. In particular, large language models (LLMs) have caught the popular imagination, because unlike advanced machine learning or natural language processing (NLP) tools, proprietary LLMs such as ChatGPT do not require technical knowledge - their user-friendly interfaces and capacity for accepting plain language as inputs democratizing access to the AI revolution for the average person.

However, existing LLM architectures are constrained by inherent limitations, such as hallucinations or information becoming rapidly outdated because of their knowledge cut-off date. To address these issues, the retrieval augmented generation (RAG) technique was designed to provide LLMs with an external data source from which to retrieve information [1]. OpenAI’s latest offering, Deep Research, extends the capabilities afforded by RAG by replacing this external data source with internet access—a potentially unlimited and real-time source from which salient information can be drawn—in addition to building on its reasoning capabilities, which ostensibly facilitates the production of a comprehensive, well-considered, and meticulously cited literature review with relatively little effort on the user’s part [2].

Social media platforms reveal polarized opinions—some abuzz with excitement about its potential, others deeply opposed. But do Deep Research tools live up to their promise? What do such tools mean for the scientific community, and in particular, the biomedical and health research ecosystem, where misinformation or missing information can directly affect clinical understanding or policy? These are no longer abstract questions - AI tools are increasingly becoming embedded in biomedical publishing workflows, grant applications, and clinical research training. In this commentary, we describe our experiences with existing Deep Research tools, their inherent limitations, and the broader implications of such technologies for the future of scientific inquiry.

## Initial Experiments: An Empirical Evaluation of Deep Research Tools

In light of the primarily anecdotal reports, we aimed to empirically evaluate the ability of currently available AI deep research tools to generate a complete review article. We selected as an exemplar a topic in which we have substantial expertise—the field of oculomics, also known as the study of the association between ophthalmic biomarkers and systemic health. This topic is particularly illustrative, as it lies at the intersection of biomedical AI and clinical translation. We assessed four leading tools: Gemini 1.5 Pro with Deep Research, Perplexity’s Deep Research mode, OpenAI’s o1 pro with Deep Research mode in February 2025, and Manus (a general AI agent built on Claude Sonnet 3.7) in March 2025.

In our experiments, we drafted an unstructured prompt that provided a general overview of the task. We used ChatGPT-4o to optimize this unstructured prompt for use in o1-pro with Deep Research mode. The inclusion of what we perceived to be the most relevant or landmark papers on the subject was variable (). To mitigate this, we specified a shortlist of essential citations to include, while encouraging the model to explore beyond this predefined set. No further prompt engineering was performed beyond the addition of these citations. These prompts are presented in Table S1, and the accompanying reviews in Table S2a-c of Multimedia Appendix 1.

We found that Gemini could deliver a well-structured output but lacked the scientific depth required of a scientific review. Perplexity’s tool encountered technical difficulties, showed poor prompt adherence, and produced an overly general overview with limited practical utility. Manus produced a review that ostensibly cited close to 300 papers, many of which were missing from the reference list, and turned out to comprise predominantly short superficial summary statements from each study with minimal elaboration or salient details. In contrast, o1-pro generated the most compelling results, providing a review that formed the basis of our structured quality assessment, which included both expert and non-expert evaluators who were asked to review the generated manuscripts independently (Textbox 1). Experts assessed quality based on citation accuracy, factual correctness, logical structure, thematic coherence, and depth of synthesis—criteria that align with standard expectations for narrative biomedical reviews. Non-experts were asked for their overall impression. The generated reviews were not edited or altered in any way prior to quality assessment.

Textbox 1. **Selected quotes from peer reviewers on the oculomics reviews generated by OpenAI’s Deep Research.**

“I think the content is generally good and they are certainly readable. It would be nice to have a less summarising review and more synthesis and critical appraisal of some of the published work. But most narrative review papers fail to do that and are not as good as this.”

“I think another shortcoming, something which is perhaps very ’human’, is the lack of any synthesis of the literature to generate new insights/future directions of research. …. essentially just paraphrasing previous articles.”

“Overall, I think Deep Research does produce a good first draft that would probably be readily accepted in a mid-tier journal, but which could be significantly improved with editing and revisions. Perhaps the outputs might improve with better prompts or guidance (eg providing refined outlines and a reference list).”

“It explained a complex, innovative topic very succinctly in a very smooth and understandable way”

Non-expert readers struggled to distinguish the AI-generated article from a traditionally authored review, highlighting the model’s ability to craft a logical, coherent, and informative narrative that was easily digestible.

Domain experts independently agreed with these general impressions. However, while they reported that the reviews seemed factually sound on the whole, they also identified several critical limitations. These included multiple instances of plausible-sounding hallucinations such as references to non-existent articles, incorrect attribution of fictitious articles to real researchers, or subtle changes in the titles of otherwise correct citations. The number of hallucinated references dropped when we included a pre-defined list of relevant references in the prompt (Table S2b to 2c in Multimedia Appendix 1), although the model elected to provide a truncated list of references only. There were several factual errors in the main text that could be easily identified with domain expertise but appeared innocuous to the non-experts (eg descriptions of non-existent prospective trials for oculomics, or erroneous claims that no AI medical devices for oculomics have received regulatory approval [3]). Finally, there was a concerning lack of citations to support claims in multiple sections (noticeably more prevalent towards the end of longer articles). This significantly undermines transparency and validation for non-domain experts, who may choose to rely on Deep Research tools as an information source based on overall impressions of reliability and seeming authenticity, regardless of whether the statements carry any epistemic weight.

Interestingly, in several instances, information was identified from pre-existing narrative reviews and paraphrased instead of reviewing the primary source, which confers risks to citation integrity. In addition, the AI tools’ inability to access paywalled literature introduces a potential bias toward open-access sources, which could affect the comprehensiveness and balance of the AI-generated output.

Overall, expert readers were impressed by the results but noted a lack of scientific depth and nuance—the elements that distinguish strong review articles from mediocre ones.

## The Death of the Narrative Review?

Therefore, despite our best efforts in crafting increasingly complex prompts to optimize the outputs, we conclude that Deep Research tools do not yet spell the end of the narrative review. There is a common misconception that writing a narrative review is relatively straightforward; readers may recall coming across a number of poorly written ones in low to mid-tier medical journals that simply list findings from prior literature. However, the art of a well-crafted narrative review lies in the thoughtful analysis of a comprehensive body of literature; beyond synthesizing key findings to form a coherent narrative, it should also provide critical appraisal, meaning, and interpretation, often accompanied by insightful commentary informed by experience and expertise, to aid in generating new insights or future directions for research [4,5].

## Broader Implications for Biomedical Research and Publishing

These critical elements are often glossed over in the pursuit of metrics for productivity and/or proxy measures for impact in academic publishing, which may incentivize the prioritization of quantity over quality and lead to a surge of low-quality reviews that dilute the scientific literature [6]. Generative AI risks exacerbating this by providing the means to automate this process. There is great irony in this. Similar to the concept of model autophagy disorder, wherein AI models trained on their own statistically simplified outputs gradually degrade over successive generations and experience a collapse in quality [7], the academic publishing ecosystem may face similar consequences when low-quality and potentially erroneous AI-generated content saturates the literature, generating a self-reinforcing cycle of mediocrity. The search for meaningful information would become akin to searching for a needle in a haystack, with the added challenge that generative AI may make the needle look more and more like hay.

Unsubstantiated claims, hallucinated information, or incomplete or inaccurate information synthesis are especially problematic in biomedical research because of the risk of influencing downstream clinical guidelines and clinical decision-making, distorting systematic reviews, and biasing early-stage research agendas. Epistemic opacity may undermine accountability by limiting researchers’ abilities to evaluate how conclusions were drawn. All these carry a risk of negative implications for patient safety, health policy development, and public trust. While methods to detect hallucinations (eg, semantic entropy) or to limit them (eg, chain-of-thought or chain-of-verification reasoning) have been developed [8,9], ultimately, hallucinations are an inherent feature of LLM architectures and probably cannot be eliminated completely [10,11], raising the question of how far can or should we trust these outputs without human oversight, however imperfect human oversight might be.

In addition, the exact method by which such tools prioritize information sources remains unclear. RAG systems may select the “optimal” information to retrieve through vector embeddings, ranking algorithms, leveraging metadata to select for data quality, or other techniques [1]. However, what does “optimal” mean in each context? How this is defined remains a “black box,” particularly for proprietary systems. This opacity presents a real challenge to the generation of verifiable, reproducible knowledge in clinical and translational research. In our experiments, we observed a tendency to prioritize commonly cited and mainstream ideas, although this improved to some extent when carefully curated references were included in the prompts. Given that English language publications from Western nations dominate the internet, Deep Research tools may be more likely to draw from these, which risks reinforcing global inequalities in biomedical research. Could unthinking overreliance on AI tools then risk introducing the danger of intellectual homogenization and potentially stifling novel, minority, or paradigm-shifting viewpoints?

The mainstream acceptance of AI in scientific training presents a fundamental challenge as well. The rise of increasingly sophisticated generative AI tools provides an easy means of outsourcing the cognitive labor of literature synthesis and critical appraisal [12], which risks eroding the intellectual rigor traditionally cultivated through research training. Descriptions of Deep Research as producing “PhD level” reviews are particularly unhelpful, as PhDs are intended to cultivate one’s ability to think critically, arguably with outputs as a byproduct of these efforts rather than the end goal. This tension is already surfacing in biomedical research and training, where increasing reliance on AI tools may reshape how we define scholarly competence. However, this may eventually become an inevitability as the narrative gradually shifts from whether AI tools should be used to how they should be used. This is reflected in the recent development of reporting checklists for transparent disclosure of generative AI in medical research—detailing the AI tools used, their roles, and their influence on research findings—which aim to safeguard reproducibility and the trustworthiness of these outputs [13].

## Knowledge vs Wisdom in the AI Era

AI tools are increasingly blurring the lines between knowledge generation and critical inquiry, but we argue that wisdom remains distinctly human. When used responsibly, as augmentations requiring careful oversight and transparent disclosure, or inspiration for epistemic humility, these tools may hold immense potential to accelerate advancements in biomedical research. However, AI cannot yet - and arguably should not - replace the critical thinking that defines human scholarship. We propose a middle path, a synergistic collaboration where the computational prowess of AI is balanced by human creativity and oversight to maintain the rigour and integrity of our scientific endeavours. We must not outsource our ability to think critically lest we lose it altogether.

## Supplementary material

10.2196/75666 Multimedia Appendix 1 Supplementary S1: Prompts used to generate the oculomics reviews. Supplementary S2a: Oculomics review generated by OpenAI’s Deep Research using a prompt with limited structured content. Reference list annotated for errors. Supplementary S2b: Oculomics review generated by OpenAI’s Deep Research using a prompt wherein the outline was pre-specified (short version). Manuscript and reference list annotated for errors. Supplementary S2c: Oculomics review generated by OpenAI’s Deep Research using a prompt wherein the outline was pre-specified (long version). Reference list annotated for errors.

## Abbreviations

AI: artificial intelligence
LLM: large language model
NLP: natural language processing
RAG: retrieval augmented generation
